# Ability of older adults to recognize cognitive changes and its relationship with mental health: a cross-sectional study

**DOI:** 10.1186/s12877-022-03096-2

**Published:** 2022-05-07

**Authors:** Hossein Ebrahimi, Mina Hosseinzadeh, Reihaneh Seifi Saray, Marian Wilson, Hossein Namdar Areshtanab

**Affiliations:** 1 Department of Mental Health and Psychiatric Nursing, Nursing and Midwifery Faculty, Tabriz University of Medical Sciences, Tabriz, Iran; 2 Department of Community Health Nursing, Nursing and Midwifery Faculty, Tabriz University of Medical Sciences, Tabriz, Iran; 3 Washington State University College of Nursing, Spokane, Washington USA; 4 Department of Mental Health and Psychiatric Nursing, Nursing and Midwifery Faculty, Tabriz University of Medical Sciences, Tabriz, Iran

**Keywords:** Aging, Mental health, Ability, Cognitive changes

## Abstract

**Introduction:**

With rising age, the incidence of physical and mental problems increases. Physiological and social changes occur across the lifespan that can affect an individual’s health and ability. The present study was aimed to determine older adult’s ability to recognize cognitive changes and its relation with mental health status.

**Materials and Methods:**

A descriptive correlational design was used to recruit 423 older adults who were referred to health centers in Tabriz, Iran in 2019 to receive primary health care. A systematic random sampling method was used for selecting participants. Data collection tools included a demographic-social questionnaire, General Health Questionnaire for assessing mental health (with 4 subscales) and a questionnaire of ability to recognize cognitive changes (with 8 sub-scales). Data were analyzed using descriptive statistics and inferential statistics.

**Results:**

The mean score of mental health of the older adults was 56.35 (8.40) which shows moderately impaired mental health. The most impaired aspect of mental health detected was the social function dimension 13.20 (2.67). The average of the total ability score was 41.19 (4.78) and the physical strength dimension had the highest average of 9.08 (1.80) and the empowerment obligation dimension had the lowest average of 3.06 (1.08). There was significant relationship between dimensions of depression (r = 0.21, *p* < 0.001), anxiety (r = 0.1, *p* = 0.04) and social functioning (r = 0.17), *p* < 0.001) with the ability to recognize cognitive changes of the older adults.

**Conclusion:**

Negative mood states and social functioning were associated with the ability to recognize cognitive changes in this population of older adults. This sample exhibited moderately impaired mental health status and relatively large burdens of untreated affective symptoms. Although causality cannot be determined with this cross-sectional design, use of social programs to keep older adults mentally active, as well as cognitive rehabilitation programs could be tested with longitudinal designs for their impact on cognitive change recognition.

## Introduction

Aging has captured the attention of experts, policymakers and public opinion for its effect on global public health and social costs. The shift in populations towards older ages started in high-income countries, yet it is now low- and middle-income countries experiencing the most significant change [1]. According to the latest census in Iran, the population of older adults has increased [1] and in 2020 about 10.3% of population were more than 60 years of age [2]. East Azerbaijan province, where the present study was conducted, is ranked sixth in Iran in older adults [3].

Older people experience both physical and cognitive changes as their age advances which can deeply affect their quality of life. In general, with rising age, the incidence of chronic diseases increases, and there is a direct relationship between aging and reduced health and ability [4]. In many studies, the words ability, empowerment and power have been used interchangeably. In a recent qualitative study on the concept of "power" among older Iranians, "awareness of personal change", "adaptation", "independence", "satisfaction", "life control" and "self-management" have been reported to describe capable and fulfilled older adults [5].

Cognitive decline is one of the important changes that occurs by aging. Early identification and initiation of treatment can prevent its progression [6]. Findings of previous studies showed that older adults can notice mental changes at the early stage of cognitive decline and they can be more aware of their cognitive decline than their family members at early stages [7]. Diseases such as dementia and depression are the most common disabilities and causes of reduced life satisfaction in the older adults [8]. People with mild cognitive impairment have more possibilities for restoration in comparison to those with advanced cognitive impairment [9]. If both the family and older adults know to receive appropriate treatment at an early stage, they can take appropriate measures to prepare for symptom progression [10].

Mental health or wellbeing is one of the most important and objective parts of empowerment, and the most effective element that creates this feeling physically and mentally in the older adults [11]. The sense of ability decreases in situations such as high stress, loss of control, loss of loved ones, the effects of aging and debilitating diseases [12]. Studies have shown that the prevalence of mental illnesses such as anxiety, depression, symptoms of psychosis, and suicidal ideation is higher among older adults than in other periods of life, which affects the management of physical illness, self-care ability, and daily activities [13, 14]. Mental health problems can have a high impact on an older person’s ability to carry out basic daily living activities, reducing their independence, autonomy and quality of life [15].

Iran as a developing country will experience the phenomenon of aging faster and in a shorter period of time in the coming years, considering the high population growth in the past decades; and this is while that sufficient infrastructure is not available [2]. Findings of a recent study indicates a high level of mental health problems in older people in Iran [11]. Scant research evidence is available to examine the ability of older adults to recognize various physical, mental, psychological, and social changes in Iran. Older age is a period of life that is associated with a decrease in mental health, yet limited studies have examined the relationship between mental health and ability of older adults to recognize cognitive changes. Our hypothesis is that mental health of older adults is associated with their ability to recognize cognitive changes. Findings of a recent study which examined mental health and cognitive functions of adults showed no effect or relation between mental health and memory in older adults [15]. Therefore, the present study aimed to investigate the ability of older adults in Iran to recognize cognitive changes and its relationship with mental health.

## Materials and Methods

This cross-sectional study with a descriptive correlational design was performed in 2019 on 462 older adults referred to health centers in Tabriz for primary health care. The sample size was determined using data obtained from a similar study [16] and considering d = 0.35, SD = 3.5, and 95% confidence interval and the standard error rate of 5% equal to 462 people.

The inclusion criteria were adults at least 60 years of age, receiving primary care at a health center in Tabriz, and not having a history of mental illness according to self-report. The research team was able to verify absence of mental illness by checking participant history in electronic medical records of the health centers. By using systematic random sampling method, 462 names were selected from a list of 169,598 who were registered in the population system of health centers. Then the selected older adults were contacted and asked to attend health centers in person to complete the questionnaires. If they did not want to attend the centers, the researcher, after coordinating by phone if they allowed, went to the door of their home and after obtaining the written informed consent completed the questionnaires by holding a folding chair to provide relative comfort for the elderly at the door. The written informed consent was obtained before completing the questionnaire. Finally, 39 samples were excluded from the study for reasons such as incorrect contact number or incorrect address and a final sample of 423 people was used for data analysis (8.4% non-response rate).

Data were collected using three questionnaires: demographic information checklist, General Health Questionnaire and the questionnaire of Ability to Recognize Cognitive Changes. The demographic information checklist consisted of 10 items including age, sex, marital status, income status, smoking status, number of children and chronic illness of older adults. General Health Questionnaire (GHQ) was used to assess the mental status with 28 questions. It consists of four sub-components, somatic symptoms, anxiety and insomnia, social dysfunction, and depression, and each component consists of 7 questions. GHQ scoring was based on the Likert scale from zero to three where lower scores indicate more impaired mental health status. The score between 0–28, 29–56 and 57–84 indicates severe impaired mental health, moderately impaired mental health and desirable mental health conditions, respectively. The validity and reliability of this tool have been reviewed and confirmed in various studies [17]. In Iran, in a recent study, Cronbach's alpha was reported to be 0.9 [18] and in the present study it was 0.88.

The questionnaire of Ability to Recognize Cognitive Changes was designed and psychoanalyzed by Tarighat [19] and consists of 25 questions that examine the ability of the older adults to recognize their cognitive changes in 8 dimensions of physical strength (5 questions), self-esteem (5 questions), spirituality (2 questions), obligation (2 questions), role performance (4 questions), position cognition (2 questions), self-management (3 questions) and self-assessment (2 questions) on a 3-point Likert scale. The range of total scores varies from 25–75 and a score of 0–25 indicates the older adults’ ability in recognition of cognitive changes is at an undesirable level, 25–50 at a relatively desirable level, and a score above 50 at a desirable level. The validity and reliability of this tool have been confirmed in a recent study and the reliability of the questionnaire through Cronbach's alpha has been reported to be 0.7 [19] and in the present study it was 0.84.

Data analysis was performed using SPSS 16 software using descriptive statistical methods such as frequency, percentage, mean and standard deviation. Normal distribution of the quantitative data was verified by the Kolmogorov–Smirnov test. Inferential statistical methods included Pearson correlations for assessing the relationship between variables. Differences between groups were examined using t tests and one way ANOVA tested for within groups differences.

## Results

In the present study 423 older adults referred to health centers in Tabriz participated. The mean and standard deviation of the age of the study participants was 68.06 (6.79) years. 51.8% of participants were female, 59.6% had at least one chronic illness, and 23.2% reported smoking. Other individual-social characteristics of the participants are given in Table 1.

The mean of mental health of the older adults was 56.35 (8.40) which shows moderately impaired mental health of participants. Among the various dimensions of mental health, the anxiety dimension had the highest mean of 14.89 (3.29) and the social function dimension had the lowest mean of 13.20 (2.67) and was the most impaired dimension of mental health.

The total score of mental health and ability to recognize cognitive changes and their dimensions are given in Table 2. The total mean score of ability was 41.19 (4.78) which indicates the ability of the older adults to recognize cognitive changes in this sample was at a relatively desirable level. Among the various dimensions of the questionnaire, the physical strength dimension had the highest mean of 9.08 (1.80) and the empowerment obligation dimension was the poorest dimension with the mean score of 3.06 (1.08).

Regarding the relationship between mental health and ability in recognizing cognitive changes, as the findings of Table 3 show, no significant relationship was determined between the total score of mental health and the total score of ability (r = 0.2, *p* = 0.27). About different dimensions of mental health, there was a significant relationship between the dimensions of anxiety (r = -0.10, *p* = 0.04), depression (r = -0.21,* p* < 0.001) and social functioning (r = 0.17, *p* < 0.001) with the total score of ability, which shows as the depression and anxiety increase, the ability of older adults to recognize cognitive changes decreases. As social function increases, the ability of older adults to recognize cognitive changes increases. Among the various dimensions of older adults’ ability in recognizing cognitive changes, dimensions of self-esteem (r = 0.17, *p* = 0.001), spirituality (r = 0.04, *p* = 0.04), performance (r = 021, *p* < 0.001), position (r = 0.32, *p* < 0.001), self-management (r = 0.33,* p* < 0.001) and self-satisfaction (r = 0.32,* p* < 0.001) had significant positive relationships with the total score of mental health.

Comparison of the total score of mental health and ability of older adults in recognizing cognitive changes according to personal and social characteristics showed that there was a significant difference in terms of physical illness (t = 4.91, *p* < 0.001), economic status (f = 3.83, *p *= 0.03), and the number of children (r = 0.06, *p* = 0.24) with the total mean of mental health indicating that better physical illness, better economic status and more number of children is associated with better mental health status. Regarding the ability to recognize cognitive changes, the variables of having children (t = 2.15, *p *= 0.03) and number of children (r = 0.16, *p* < 0.001) showed a significant difference with the mean score of ability to recognize cognitive changes. In participants with more children, there was a greater ability of older adults to recognize cognitive changes. (Table No. 4).

## Discussion

This study aimed to determine the ability of older adults to recognize their own cognitive changes and its association with mental health. The findings showed that the ability of older adults to recognize cognitive changes is at a relatively desirable level within the study population and the mental health status of participants shows moderately impaired mental health. Regarding the relationship between mental health and ability to recognize cognitive changes, no significant relationship was determined between the total score of mental health and the total score of ability of recognition. Yet, the mental health sub-scales of anxiety, depression and social functioning were all significantly associated with the total score of ability indicating these dimensions may be most important in recognizing cognitive changes. These findings are important because they can identify vulnerable populations who may need more proactive screening for cognitive changes.

Although the explanation for our findings are not fully known, one possible mechanism for the association between depression and cognitive disorder is a reflection of an underlying common cause such as cerebrovascular disease. When depression occurs it causes structural changes to central nervous system (CNS) white matter which can decrease data processing speed, poor memory and executive dysfunction as well as increased likelihood for progression to dementia [20]. These changes could alter the aging person’s ability to recognize the onset of cognitive decline. Anxiety could similarly alter cognitive processing and the absence of social networks to help point out cognitive changes [21] could explain associations we detected as well.

In a recent study in Iran, it was reported that the older adults had moderate level of mental health, and depression and social dysfunction were the most impaired dimensions [22], which is somewhat consistent with the findings of the present study. The dimension of depression had a low score in the present study. Depression is a serious health problem and a major public health concern among older adults [23]. In a recent meta-analysis study in Iran, the prevalence of depression among Iranian elderly is reported to be 43% and 38% of the elderly suffer from mild depression [24]. Chronic disease, disability, lack of social support and socio-economic status are documented as the risk factors of late life depression [23]. A recent study showed that more than 80% of the older adults had limitations in their functions [24]. In our study the dimension of social functioning was the poorest dimension of mental health among older adults. Findings of another recent study reported that social dysfunction had the greatest impact on older adults' perception of mental health [25]. All of these findings underscore the importance of assessing for mental health and social support in older adults and providing strategies to assist whenever possible.

In the present study the ability of older adults to recognize cognitive changes is at relatively desirable level on average. However, according to the cut of point of the scale of ability to recognize cognitive changes, it is not in completely favorable level, indicating an opportunity to intervene. Findings of a recent study in Japan showed that older adults have relatively high ability to recognize their own cognitive decline at early stages. This is somehow consistent with our findings [7]. A similar study was not found using the identical scale, but some aspects of the scale were used, for example self-management ability; the findings of a recent study showed that the self-management ability of the older adults is in moderate level on average, which is consistent with the findings of the present study [26]. Unique to our study was the measurement of ability to recognize cognitive changes, which has not been widely studied in relationship to negative mood stated. The most similar studies to date have examined perception of cognitive decline [27]; while a similar concept, there are some subtle differences in that one's perception and ability may not be precisely the same thing. Our findings do add some validation to the literature on using ability to recognize cognitive changes as a measurement, in that it did perform similarly to the perception measurements. Future research could compare these two concepts more intentionally and add to their validity testing.

About the association between decline the ability to recognize cognitive changes and some dimensions of mental health, literature shows that combined depression and cognitive dysfunction is reported to be present in about 25% of older adults. A more recent study reported that in the onset of late life depression it is noticeable decline in older adult’s cognition ability and their function [28]. It was shown that having common psychiatric disorders such as anxiety and depression (not disorders such as psychosis, which are classified as major mental disorders) are in significant relationship with disability and reduced cognition recognition ability [29]; this is consistent with our findings. In a recent longitudinal study findings indicate that having mild psychological problems such as depression, anxiety, social dysfunction, and lack of self-confidence in old age is associated with the decreased cognitive ability [30]. According to the findings of a recent study the prevalence rates of subjective cognition impairment was ranging from 15 to 50% across studies and it is reported that it was commonly co-occurring with depression and anxiety [27]. In the present study, among the dimensions of older adult’s ability, a significant relationship was observed between self-management and self-satisfaction with mental health. Some recent studies have reported a strong significant relationship between self-management in the older adults and their sense of well-being and social functioning [28].

The importance of our findings are that known interventions can be implemented early on if cognitive changes occur, which can, potentially, ease burdens on society and costs of care. As the population ages, it is also essential to plan strategies that might minimize additional costs. Knowing that older adults with depression, anxiety and poor social function may be less likely to identify their own cognitive changes means that more effort will be needed in assessments. Routine or universal screenings could be added that would detect problems early on and direct health care providers or caregivers of older adults to potential remedies. Additional social supports can be put in place, as simple as making family members aware of the need to check in more often on the older adults' well-being and be sure the home environment is safe.

One of the limitations of the present study is the self-reporting nature of the data, which due to the older age of the participants as well as their mental state at the time of completing the questionnaire may affect the accuracy of the collected data. Another limitation of our study was non-response rate of 39 subjects (8.4%) of the sample which may affect our findings. The cross-sectional design does not allow for any cause-effect conclusions, yet can direct further hypothesis testing. Because our sample is restricted to one city and we do not have comparison data available to determine how well our sample matches the general Tabriz City population, it is possible our findings would not be generalizable to the larger city population or to other populations.

## Conclusion

Negative mood states and social functioning were associated with the ability to recognize cognitive changes in this population of older adults. This sample exhibited moderately impaired mental health status and relatively large burdens of untreated affective symptoms. Although causality cannot be determined with this cross-sectional design, use of social programs to keep older adults mentally active, as well as cognitive rehabilitation programs could be tested with longitudinal designs for their impact on cognitive change recognition. Codified programs for the diagnosis and treatment of common mental disorders in old age are recommended.

**Table 1:** Demographic characteristics of study participants (*N* = 423)

*N* (%)		Variables	*N* (%)	Variables	
252(59.6)	**yes**	**Physical illness**	204(48.2)	male	Gender
171(40.42)	**no**		219(51.8)	female	
98(23.2)	**yes**	**Smoking** **experiences**	2 (0.5)	Single	**Marital status**
325 (76.8)	**no**		347(82)	Married	
			74(17.5.1)	Divorced/widow	
68.06 (6.79)		Age **	241 (57)	Expenditure < earnings	Economic status*
5.10(2.13)		Number of children**	170 (40.2)	Expenditure = earnings	
			11(2.6)	Expenditure > earnings	

^*^Note: missing data from 1 participant, **Note: Mean (*SD*)

**Table 2:** - The average score of mental health and ability and its dimensions in the older adults referring to health centers in Tabriz

Dimensions of mental health questionnaire	Mean (SD)	Minimum (observed)	Maximum (observed)
Physical strength	14.74 (2.96)	7	48
Anxiety	14.89 (3.29)	7	28
Social performance	13.20 (2.67)	7	34
Depression	13.51 (2.66)	7	25
**Total mental health scor**	56.35 (8.40)	30	99
**Dimensions of the Ability to recognize cognitive changes**			
Physical strength	9.08 (1.80)	5	18
Self-esteem	8.08 (1.59)	3	18
Spirituality	3.27 (1.22)	2	5
Empowerment obligation	3.06 (1.08)	2	8
Performance	5.67 (1.55)	2	10
Situation	3.27 (1.09)	2	10
Self-management	5.09 (1.50)	2	12
Self-satisfaction	3.64 (1.16)	2	6
**Total Ability Score**	41.19 (4.78)	30	57

**Table 3:** Association between mental health and its dimensions with ability and its dimensions in the older adults referring to health centers in Tabriz

	**Total mental health score**		**Depression**		**Social function**		**Anxiety**		**Physical ability**	
**The ability of older adults **	r	p	r	p	r	p	r	P	r	p
**Physical ability**	-0.01	0.78	-0.09	0.05	0.04	0.36	0.03	0.44	0.03	0.49
**Self-esteem**	0.83	0.01	0.08	0.07	0.12	0.01	-0.10	0.03	0.17	0.001
**Spirituality**	0.04	0.04	0.05	0.27	0.06	0.20	-0.12	0.01	0.07	0.15
**Empowerment obligation**	-0.01	0.83	0.09	0.06	-0.04	0.38	0.06	0.16	0.03	0.43
**Role Performance**	0.21	<0.01	-0.20	<0.001	0.08	0.08	0.05	0.29	0.07	0.14
**position cognition**	0.32	<0.001	-0.23	<0.001	0.39	<0.001	-0.04	0.33	0.01	0.07
**Self-management**	0.33	<0.001	0.32	<0.001	0.23	<0.001	0.08	0.09	0.01	0.75
**Self-satisfaction**	0.32	<0.001	0.33	<0.001	0.12	0.01	0.08	0.07	0.11	0.01
**Total ability Score**	0.20	0.27	-0.21	<0.001	0.17	<0.001	0.10	-0.04	0.09	0.08

: Significant relationship (*p*<0.05)

**Table 4:** Differences in mental health score and empowerment score by demographic variables

**Variable**		**Mental health score**		**Ability score**	
		**Mean(SD)**	**Test**	**Mean(SD)**	**Test**
**Gender**	**Male**		t=0.71 Df=421 P=0.47	47.30(4.97)	t=0.23 df=380 P=0.81
	**Female**	56.8 (4.13)		(4.60)47.18	
**Marital status**	**Single**	59.4 (50.94)	F=0.15 Df=3 P=0.92	(3.12)47	f=0.70 df=3 P=0.54
	**Married**	56.8 (28.31)		(4.49)47.06	
	**Divorced/Widow**	58.9 (50.19)		(6.10)48.31	
**Physical illness**	**Yes**	7.66))44.52	t=4.91 df=371 *P*=<0.001	47.10(5.01)	t=-0.78 df=386 *P*=0.43
	**No**	(8.75)40.19		(4.03)47.50	
**Smoking experiences**	**Yes**	(9.16) 43.10	t=0.05 df=371 *P*=0.95	46.22(4.12)	t=-1.65 df=386 *P*=-1.65
	**No**	8.15))43.04		47.38(4.80)	
**Having children**	**Yes**	(8.20) 43.55	t=1.41 df=333 *P*=0.15	47.16(4.70)	t=2.15 df=373 *P*=0.03
	**No**	(8.35)41.60		43(4.09)	
**Economic status**	Expenditure< earnings	(9.41) 43.49	f=3.83 df=2 *P*=0.03	47.17(4.84)	f=0.03 df=2 *P*=0.96
	Expenditure= earnings	41.52(6.41)		47.05(4.91)	
	Expenditure> earnings	43.84(10.46)		47.26(2.46)	
**Age**	r= 0.24, *p*=0.06			R=0.06, *p*=0.24	
**Number of children**	r= 0.16, *p*<0.001			R= 0.16, *p*<0.001	

: Significant relationship (*p*<0.05)

## Data Availability

The datasets generated / analyzed during the current study are not publicly available due to ethical concerns but are available from the corresponding author on reasonable request. (Ethical committee of Tabriz University of Medical Science have restrictions about availability of data).
